# Supplementary material for: Genome-wide identification and characterization of FAD family genes in barley
Source: PeerJ. 2024 Feb 29;12:e16812. doi: 10.7717/peerj.16812 (PMC10909363; doi:10.7717/peerj.16812)
Supplement: Supplemental Information 1 — All the pictures in the article are analyzed using the protein sequences of 24 FAT members screened from barley. [file peerj-12-16812-s001.doc]

HORVU.MOREX.r3.2HG0101440.1

MAALLLLKQPCRPAARTWTTRTRNDTALQPVTSITYLRTGRSAAGAVKLEEGGTDDEWLAYLEPAKLEVFDQLEPWAEANVVPLLKPAEVAWQPSDLLPDPASLGADGFHAACCDIRARAACLPDAHLVCLVGNMVTEEALPSYQSMANRFEAVHDLTGSSGTAWARWTRGWSAEENRHGDVLNRYLFLSGRVDMPQVERTIHNLINSGMVLKAARSPYHGFIYVAFQERATSISHGNMARRAKEHGDLALARICGAIAADEKRHELAYTRIVGKLFEVDPDGAVRALAYMMRRRIVMPASLMNDGRDGDLFAHYGAVAQQAGIYTASDYRSILEHLIKQWGVEELAAAGLSDEGRRARDYVCALPQKIRMLEEKAHQRSAQKAQPIKSASFSWIFDRPVRITMA

HORVU.MOREX.r3.2HG0101470.1

MAALLLLKQPCRPAARTWTTRTRNDTALQPVTSITYLRTGRSAAGAVKLEEGGTDDEWLAYLEPAKLEVFDQLEPWAEANVVPLLKPAEVAWQPSDLLPDPASLGADGFHAACCDIRARAACLPDAHLVLQRHRMGALTRGWSAEENRHGDVLNRYLFLSGRVDMPQVERTIHNLINSGMVLKAARSPYHGFIYVAFQERATSISHGNMARRAKEHGDLALARICGAIAADEKRHELAYTRIVGKLFEVDPDGAVRALAYMMRRRIVMPASLMNDGRDGDLFAHYGAVAQQAGIYTASDYRSILEHLIKQWGVEELAAAGLSDEGRRARDYVCALPQKIRMLEEKAHQRSAQKAQPIKSASFSWIFDRPVRITMA

HORVU.MOREX.r3.2HG0101500.1

MAALLLLKQPCRPAARTWTTRTRNDTALQPVTSITYLRTGRSAAGAVKLEEGGTDDEWLAYLEPAKLEVFDQLEPWAEANVVPLLKPAEVAWQPSDLLPDPASLGADGFHAACCDIRARAACLPDAHLVCLVGNMVTEEALPSYQSMANRFEAVHDLTGSSGTAWARWTRGWSAEENRHGDVLNRYLFLSGRVDMPQVERTIHNLINSGMVLKAARSPYHGFIYVAFQERATSISHGNMARRAKEHGDLALARICGAIAADEKRHELAYTRIVGKLFEVDPDGAVRALAYMMRRRIVMPASLMNDGRDGDLFAHYGAVAQQAGIYTASDYRSILEHLIKQWGVEELAAAGLSDEGRRARDYVCALPQKIRMLEEKAHQRSAQKAQPIKSASFSWIFDRPVRITMA

HORVU.MOREX.r3.2HG0106740.1

MSTLQSSLLMCPLARPWTRRGNNRGLQQVLTNITYWRCSSAGDGGRFMAEIAGLGKGSTGTGRRAAVAAKHEEEGTDDEWLMYLKPAKLEVFHHLEPWAEANVVPLLKPAEVAWQPTDLLPDLASLGADGFHAACSDITARAAGLPDAHLVCLVGNMVTEEALPTYQSIPNRFEGVRDLTGSSGTAWARWIRGWSAEENRHGDVLNRYLFLSGRVDMRQVERTIHNLINSGMVMNAARSPYHGFIYVAFQERATSISHGNTARRAKEHGDMALARICGAIAADEKRHELAYTRIVGKLFKIDPDGAVRALAYMMRLRIVMPASLVTDGRDDDLFAHYAAVAQQAGIYTASDYRSVLEHLIKQWGVEELVAGELSDEGRRARDYVCALPRKIRRLEEKANLRNGRKAQPMTPVSFSWIVDRPINITIG

HORVU.MOREX.r3.2HG0106840.1

MKCTLLRIVLACRRCSSAGDGGRFMAEIAGLGKGSTGTGRRAAVAAKHEEEGTDDEWLMYLKPAKLEVFHHLEPWAEANVVPLLKPAEVAWQPTDLLPDLASLGADGFHAACSDITARAAGLPDAHLVCLVGNMVTEEALPTYQSIPNRFEGVRDLTGSSGTAWARWIRGWSAEENRHGDVLNRYLFLSGRVDMRQVERTIHNLINSGMVMNAARSPYHGFIYVAFQERATSISHGNTARRAKEHGDMALARICGAIAADEKRHELAYTRIVGKLFKIDPDGAVRALAYMMRLRIVMPASLVTDGRDDDLFAHYAAVAQQAGIYTASDYRSVLEHLIKQWGVEELVAGELSDEGRRARDYVCALPRKIRRLEEKAHQRRKAKPTTLTPFSWIFDKPVNITMA

HORVU.MOREX.r3.2HG0108020.1

MARLLLPQCCCGLTPLPRPRRAVALPPSPALLASAAPRRALSLRVAVAAPTRLVTAEDDGSGSSSRAAAGQDGGEDPAAGFDPGAPPPFGLADIRAAIPKHCWVKDPWRSMGYVVRDVVVVLALAAAAARLDSWLAWPVYWAAQGTMFWALFVLGHDCGHGSFSNNPKLNSVVGHILHSSILVPYNGWRISHRTHHQNHGHVEKDESWHPLPEKLYRSLDSSTRKLRFALPFPMLAYPFYLWSRSPGKSGSHFHPSSDLFQPNEKNDILTSTTCWLAMAGLLAGLTAVMGPLQILKLYAVPYWIFVMWLDFVTYLHHHGHNDKLPWYRGKAWSYLRGGLTTLDRDYGWLNNIHHDIGTHVIHHLFPQIPHYHLVEATEAAKPVLGKYYREPDKSGPFPFHLFGALARSMKSDHYVSDTGDIIYYQTDPKLAAGAHTSD

HORVU.MOREX.r3.2HG0129520.1

MATASGLSAPLQLLSLRRAPTKNLSPRRAAVGALSGSLVVKRFSLCNGRSHHQFLPLKQRGRLQAAVLPVTPPLLDDEDKRKQMSEDYGFKQVGEQLPDSVTLKDVMDTLPKEVFEIDDVKAWASVLISVTSYAFGLFLISKAPWYLLPVAWAWAGTAVTGFFVIGHDCAHKSFSRNKLVEDIVGTLAFLPLIYPYEPWRFKHDRHHAKTNMLVEDTAWQPVWQKEIESSSFLRKAIIFGYGPIRPWMSIAHWLMWHFDLKKFRPNELPRVKISLACVFAFMAIGWPLIILQSGIAGWFKFWFMPWMVYHFWMSTFTMVHHTAPHIPFKSSKEWNAAQAQLNGTVHCSYPRWIEILCHDINVHVPHHISPRIPSYNLRAAHDSIKQNWGKYINEASWNWRLMKTILTTCHVYDKERYYVSFDELVPEESQPIRFLKKFMPDNA

HORVU.MOREX.r3.2HG0161410.1

MAFRACLPSHKASPSPSVAQRRAGNGPPPVVAMASTINEVKTAKKPYAPPREVHLQVMHSLPAQKQEIFDSLQSWARDNLLNLLKPVEKSWQPQDFLPDPSSEGFYDEVKELRERAKEIPDDYFVCLVGDMVTEEALPTYQTMLNTLDGVRDETGASPTAWAVWTRAWTAEENRHGDLLNKYMYLSGRVDMRQIEKTIQYLIGSGMDPGTENNPYMGFLYTSFQERATFISHGNTARHAKQFGDLKLAQICGTIAADEKRHETAYTKIVEKLFEIDPDYTVLAFADMMRKKISMPAHLMYDGEDDNLFEHFSSVAQRLGVYTAKDYADILEFLVQRWKVADVTGLSGEGRRAQDYVCTLATRFRRLDERAQARAKQGPVVPFSWVYDRKVQL

HORVU.MOREX.r3.2HG0183780.1

MGATRADAGDKEEGVMATDFFWSYTDEPHASRRREILAKHPQIKELFGPDPLAFIKIAVVVSLQLWTATLLRDAGWSKILPVAYFFGSFLNHNLFLAIHELSHNLAFTTPSLNRWLGIFANLPIGVPMSVTFQKYHLEHHRFQGVDGIDMDIPSQTEAHVVKNAVSKSVWVVLQLFFYALRPLFLKPKPPGLWEFTNLTIQLALDAAMVYLFGWKSLAYLILSTFLGGGMHPMAGHFISEHYVFSPEQETYSYYGPLNLMTWHVGYHNEHHDFPRIPGAKLHKVKEIAPEYYNSLKSYRSWSQVIYMYVMDQTVGPFSRMKRKAPKKDS

HORVU.MOREX.r3.3HG0288610.1

MSSIYVGALELRAMLKELVGFGARRPGSDADVPECLRGWAADADAGSLSERVTASACDVSLRDDRETLMATARVELVCLVCNMVHVHGQRRRRCRRSTCAWATGWRASATTRAATSSHWRSGFRVDMRQVERTVHHLLRNGMYMLMPSIPYHKVIYGSFQDRATFICHTHTAKQAAHHGARYLVKICGIIAADEKRHQMTYTKEAAKLFELNPQGMVRALTIVLRDKITMHDQLMTDDHEADLFDNFSAVVQRTGVYTARDYGDMVEHFVRRWELADLAREQLSGEGRCAQQYVFLFFFENRAQEYVCGLPHKIRREEQLARDRASVWLAGSMRPIFMF

HORVU.MOREX.r3.3HG0307490.1

MQAQSILRVPGHPAAALPLPRRQCRRVSAVAAAPSVQRGVTHSMPPEKAEVFQSLRGWAGSSLLPLLRPVEDIWQPADFLPDSSSEMFEHEVAELRARAAALPDEYFVVLVGDMVTEEALPTYQTMINTLDGVRDETGASACPWAVWTRAWTAEENRHGDVLNKYMYLSGRVDMRMVEKTVQYLIGSGMDPRTENNPYLGFVYTSFQERATAVSHGNTARLAKAHGDDVLARACGTIAADEKRHETAYSRIVEQLLRLDPDGAMLAIADMMRKRITMPAHLMHDGSDMDLFEHFASVAQRLGVYTAQDYTDIVEFLVKRWKLETLEGGLSGEGRRARDFVCGLAPRMRRAAERAADRARKDEPRKVKFSWIFDREVVV

HORVU.MOREX.r3.3HG0309010.1

MAKTGPAACTSAKICFFFSNKSDAGSGRATRLGWMLRPANSGCSLATRWTAAAAAAVEAPARSIDVGFAPVPREQAEMVQSLNGWVAENMLPLLSPVESSWQPHDFLPCSAAPPGASEEEALSAFMEGVAALRAGAAGVPDEVLVCLVGNMVTEEALPSYQSMGNRTEGIADDTGASGLPWAQWLRGWTAEENRHGDLLNRYLYLSGRVDMRQVETTVHHLLRNGMEMLVPKSPYHSVIYGAFQERATFVSHVNTARLAGQHGDQALAKICGVIAADEKRHEAGYTRVSAKLFEVDPDGIVRALAHVMRGKVTMPGLLMSDGRDAESSLFDRFSAVAQRAGVYTARDYGDLVEHFVRRWRVAELAGLSGEGRRAQEYVCGLPPKIRRMEELAHQKAARSELRPARFSWIFGRHVMVG

HORVU.MOREX.r3.3HG0310210.1

MASRMALRPHAVTPPLSAAGRAARRTGSLRVLAVASSASASAKVESKKTFAPPKEVHVQVTHSMPPQKMEIFQSLDGWARDNLLLHLKPVEKCWQPQDFLPDPASDGFHDEVKELRERAKEIPDDYLVCLVGDMITEEALPTYQTMLNTLDGVRDETGASPTAWAVWTRAWTAEENRHGDLLNKYLYLTGRVDMRQIEKTIQYLIGSGMDPRTENNPYLGFIYTSFQERATFISHGNTARHAKDFGDLKLAQICGIIASDEKRHETAYTKIVEKLFEIDPDGTVLALADMMRKKIAMPAHLMFDGQDEKLFDHFSMVAQRLGVYTARDYADILEFLVGRWKVPELTGLSGEGHKAQDYLCTLAGRIRKLDERAQSRAKQAGKMPFSWVYGREVQM

HORVU.MOREX.r3.4HG0354970.1

MGAAARRAPEQEQSCKATEDFDAAKPPPFRIGDVRAAVPAHCWRKSPLRSLSYVARDVAVVAALAVVAWWLNSWAVWPLYWAAQGTMFWALFVLGHDCGHGSFSDSLTLNSVVGHLLHTFILVPYNGWRISHRTHHQNHGHIDKDESWHPITENVYKEMEPSTKKLRFSLPYPLLAFPVYLWYRSPGKNGSHFNPSSDLFSPKERRDVIVSTTCWFTMIALLIAMACVFGPVPVLKLYGVPYAVFVMWLDLVTYLHHHGHQDLPWYRGEEWSYLRGGLTTVDRDYGWINNIHHDIGTHVIHHLFPQIPHYHLVEATKAARPVLGRYYREPEKSGPLPLHLFHVLLRSLRADHFVSDVGDVVFYQTDPSLNGDNWTKNGKHK

HORVU.MOREX.r3.4HG0384980.1

MARLALSDCRGLTPLRARSAIALPSPPHLAAGPRRPAPAAIHRDWALRVSAPTRLASVLEEDKRGLGGAEEAGSSSAGFNPGAPPPFGLAEIRAAIPKHCWVKDPWRSMSYVLRDVLVVLGLAAAAARADSWLVWPLYWAAQGTMFWALFVLGHDCGHGSFSSNPKLNSVVGHILHSSILVPYNGWRISHRTHHQNHGHVEKDESWHPLPQRLYNSLDSMTKKLRFSMPFPMLAFPLYLFARSPGKEGSHFNPNSDLFQPNEKKDVLTSTASWLAMIGVLAGLTFVMGPLKMLKLYAIPYVIFVMWLDFVTYLHHHGHEDKVPWYRGKEWSYLRGGLTTLDRDYGLINKIHHDIGTHVIHHLFPQIPHYHLVEATEAAKPVLGKYYKEPEKSAPLPFHLLQVLSRSLKKDHYVSDTGDIVYYQSESETSTSGQRSD

HORVU.MOREX.r3.5HG0457600.1

MYLMACMPSCGTFHAPLVSSCLRRKFTVVATASKAKVGTPGKAFTQVQFAQPFPPEKKEVFDSLERWAEDNILVLLKPVEKSWQPQDYLPDPSSDGFYDEVKELRERAKEIPDDYLVCLVGDMVTEEALPTYQTMLNILDGGVGDDTGTSPASWAVWTRAWTAEENRHGDLMNKYMYLTGRVDMRQIEKTIQYLLGAGMDPKTEGNPYQGYIYTSFQERATFISHGNTARHARKYGDLKLAQVCGTIAADEKRHETAYTKIVEKLFEVDPDYTVLAFAAMMRKKVTMPAHLMYDGQDDNLFEHFSAVAQRLGVYTAMDYADILEFLVQRWNVANLTGLSGEGRRAQDFLCSLGPRFRKLEERAQGRAKQLPVVPFSWIHGRQVQL

HORVU.MOREX.r3.5HG0466020.1

MAPAMRPEQEASCKATEDHRRSEFDAAKPPPFRIGDVRAAVPPHCWRKSPLRSLSYVARDVAVVAALAAAAWRADTWALWPLYWAVQGTMFWALFVLGHDCGHGSFSDSGTLNSVVGHLLHTFILVPYNGWRISHRTHHQNHGHIEKDESWHPITEKVYQKLEPRTKTLRFSVPFPLLAFPVYLWYRSPGKEGSHFNPSSDLFTPKERRDVIISTTCWFTMIALLIGMACVFGLVPVLKLYGVPYIVNVMWLDLVTYLHHHGHQDLPWYRGEEWSYLRGGLTTVDRDYGWINNIHHDIGTHVIHHLFPQIPHYHLVEATKAARPVLGRYYREPEKSGPLPMHLITVLLKSLRVDHFVSDVGDVVFYQTDPSLSGDNWAGTDKQK

HORVU.MOREX.r3.5HG0474290.1

MARTGLADATAPDADAMPAATKDAADVRMISTKELQAHAAADDLWISISGDVYDVTPWLRYHPGGEVPLITLAGQDATDAFMAYHPPSVRPLLRRFFVGRLSDYTVPPASADFRRLLAQLSSAGLFERVGHTPKFLLVAMSVLFCVALYCVLACSSTGAHMFAGGLIGFIWIQSGWIGHDSGHHQITSHPALNRLLQVVSGNCLTGLGIAWWKFNHNTHHISCNSLDHDPDLQHLPLFAVSTKLFNNLWSVCYERTLAFDAISKFFVSYQHWTFYPVMGFARINLLVQSIVFLITQKKVRQRWLEIAGVAAFWVWYPLLVSCLPNWWERVAFVLASFVITGIQHVQFCLNHFSSAVYVGPPKGNDWFERQTAGTLDIKCSPWMDWFHGGLQFQVEHHLFPRLPRCHYRMVAPIVRDLCKKHGLSYGAATFWEANVMTWKTLRAAALQARDATTGSAPKNLVWEALNTHG

HORVU.MOREX.r3.5HG0486420.1

MPGLITLPATLNQQGSFYLCSTEKMSMLKPFPRGLAMPAQAHPTWCRSRAAARAGRWACKVAATANFEGTVTGMAAQEQAEAEVVRCLSRSGWVEEQLLPLLTPVEDAWQPSDLLPCFSLSAAGLSAEQQPAMTMTTQELQAQASGVPDDVLVCLVGNMVTEEALPTYMCMGNRVPGFRDDTGCSDLPWARWLRGWTAEENRHGDLLNRYLYLSGRVDMRQVERTVHHLLRNGMQMLRPSSPYHNAVYGSFQERATFISHTHTAKHAARHGDRCLAKICGVVAADEKRHETAYTKVAAKVFELDPDGMVRALAAVLQDKITMPGQLMTDGRDADLFDHFSAVAQRTGVYTARDYGDMVEHFVRRWKVADLAGGQLSGEGRRAQEYVCGLPRKIRRVEELAHDRAIKAAKEPEFGRFSWVFDRSVCIRT

HORVU.MOREX.r3.5HG0535350.1

MHLTAPLPSCRAFQAPLWCSCLRSKITVMATASKAKDGVPGMAFSPRKAPHRDRVAHTLPPEKREIFDSLNSWAEDNLLVLLKPVERSWQPQDYLPDPSLDRFYDEVKELRERAEEIPDDYLVCLVGDMVTEEALPTYQKMLNILDGGVRDETGSSPTSWAIWTRAWTAEENRHGDLMNKYIYLTGRVDMRQVEKTIQYLVGAGMDPRTEANPYEFFIYTSFQERATFISHGNTARHARKYGDQKLAQICGTIAADERRHELAYTKIVEKLFEVDPDYTIQAFASIMKKKITMPAHLMYDGEEDNLFEHFSAVAQRLGVYTAVDYADILEFLIQRWNVAGLIGLSGEGRRAQDYLCSLGPRFRKLVERGQGSGKQLPVVPFSWIYGRQVQL

HORVU.MOREX.r3.6HG0609520.1

MGAGGRMTEKEREKQEQLGRAGGGAAFQRSPTDKPPFTLGQIKKAIPPHCFQRSIIKSFSYVVHDLVIIAALLYAALVWIPTLPTVLQLGAWPLYWIVQGCVMTGVWVIAHECGHHAFSDYSLLDDTVGLVLHSWLLVPYFSWKYSHRRHHSNTGSLERDEVFVPKQKEALAWYTPYIYNNPIGRLVHIVVQLTLGWPLYLALNASGRPYPRFACHFDPYGPIYNDRERAQIFISDVGVLAVSLALLKLVSSFGFWWVVRVYGVPLLIVNAWLVLITYLQHTHPALPHYDSTEWDWLRGALATMDRDYGILNRVFHNITDTHVAHHLFSNMPHYHAMEATKAIKPILGEYYQFDGTPVAKATWREAKECIYVEPEDRKGVFWYSNKF

HORVU.MOREX.r3.6HG0609610.1

MGAGGRMTEKELRGRTGAVEIFERAPSDKPAFTLAQIKKAIPPHCFQRSVIMSFSYVVYDLVMVASLLYAALVWIPALPIMQQLGAWPLYWFVQGCVMTGIWVIAHECGHHAFSDYLLLDNMVGLVLHSCLLVPYFSWKYSHRRHHANTGSMENDEVYVPKKKEALPWYTPYIYNNPVGRLGYIVVQLTLGWPMYLALNTSGRTYPRFVCHYDPYGPMYSDMERAQVFVSDVGVLAVSLGLLKLVWAFGFWWVMRVYGVPLLVVNAWLVVITYLAHTHPALPHYDSTEWEWLRGALATMDRDLGVLNRVFHNTTDTHVAHHLFSSIPHYHAMEATKAIRPVLGDYYQLESNPIARATWRSAKECIYVQPEDRKGVFWYTNKF

HORVU.MOREX.r3.6HG0609630.1

MGVGGRMTEQEREKQELLGRPTFERIPTDKPPFTLGQIKMAIPPHCFQRSLIKSSSYLVHDLVIIAALLYAALVWIPALPSMLQLGAWPLYWVAQGCVMFGVWVIAHECGHHAFSDYSLLNDIVGLVLHSWLLVPYFSWKHSHRRHHSNTGSLERDEVFVPRPKEALPWYTPYIQNSSVVRVVLIIVQLTLGWYMYLSLNTWGRPYSRFACHFDPYSPIFNDRERAQIFLSDVGVLAVSFAMLKLVSTFGFWWVMRVYGVPLMIVNAWLVLVTYLHHTHQALPHYDSTEWDWLRGALATMDRDYGIILNRVFHNITDTHILHHLYSNIPHYHAMEATKAIKPILGEYYQIDRTPLAKATWREAKECLYIEREDNKGIFWYSNKF

HORVU.MOREX.r3.7HG0668240.1

MSTAAAAAAATAVTMSPRSTGYPSSCKPSNAGCYCKPPAAATISTLRCRSAVSTKGGPTAGRREEEEEWRRYLAPERLEVLAQLEPWAEANMLPLLKPADEVWQPADMLPDAAALGADGFHEACLDLRARAEGVPDAQLVCLVGNMVTEEALPTYQSMSNRFEGTRDATGADGTAWARWIRGWSAEENRHGDVLSRYMYLSGRLDMRQVERTVHRLISSGMAMHAPASPYHGFIYVSFQERATAISHGNTARQVRAHGDAALARICGAIAADEKRHEAAYTRVVAKLFEVDPDAAVRAMAYMMRRRITMPAALMDDGRDADLFAHYAAAAQQAGVYTASDYRGILEHLIRQWRVEELSAGLSGEGRRARDYVCALPDKIRRMEEKAHDRVRKEPTPVPFSWIFDRPVSVVLH
