# Supplementary material for: Genome-wide identification and characterization of FAD family genes in barley
Source: PeerJ. 2024 Feb 29;12:e16812. doi: 10.7717/peerj.16812 (PMC10909363; doi:10.7717/peerj.16812)
Supplement: Supplemental Information 2 — Each gene corresponds to a pair of primers for qRT-PCR [file peerj-12-16812-s002.doc]

**Data S1 The specific primers for qRT-PCR**

| Gene Number | qRT-PCR |
| --- | --- |
| HvFAD8 | CAAGGTGCACACGTAGTCCT  TGACTACACGGTGCTTGCAT |
| HvFAD13 | GGAACTCAGAGAACGTGCCA  GGGGCTAGCACCTGTTTCAT |
| HvFAD14  HvFAD15  HvFAD21  tublin | AGCTTCTCGGACAGCTTGAC  AGCGTAGCTTCTTGGTGCTT  CCCGTAGTCCCGATCAAGTG  TCTGACCTTTGTGATGGGGC  AGTTTGGCGTGTGTCTTTGC  CCTGTGCAGCATTCCACTCT  AGTGTCCTGTCCACCCACTC  AGCATGAAGTGGATCCTTGG |
