# Supplementary material for: Genome-wide identification and characterization of FAD family genes in barley
Source: PeerJ. 2024 Feb 29;12:e16812. doi: 10.7717/peerj.16812 (PMC10909363; doi:10.7717/peerj.16812)
Supplement: Supplemental Information 3 — HvFAD14 homologous recombinant primer for subcellular localization. [file peerj-12-16812-s003.doc]

**Data S3 The specific primers for Subcellular localization**

| Gene Number | Subcellular localization |
| --- | --- |
| HvFAD14 | gtcgacggtatcgataagcttATGGGCGCCGCGGCGAGG  tcccccgggctgcaggaattcTCACTTGTGCTTGCCATTTTTC |
|  |  |
