# Supplementary material for: Genome-wide identification and characterization of FAD family genes in barley
Source: PeerJ. 2024 Feb 29;12:e16812. doi: 10.7717/peerj.16812 (PMC10909363; doi:10.7717/peerj.16812)
Supplement: Supplemental Information 5 [file peerj-12-16812-s005.pdf]

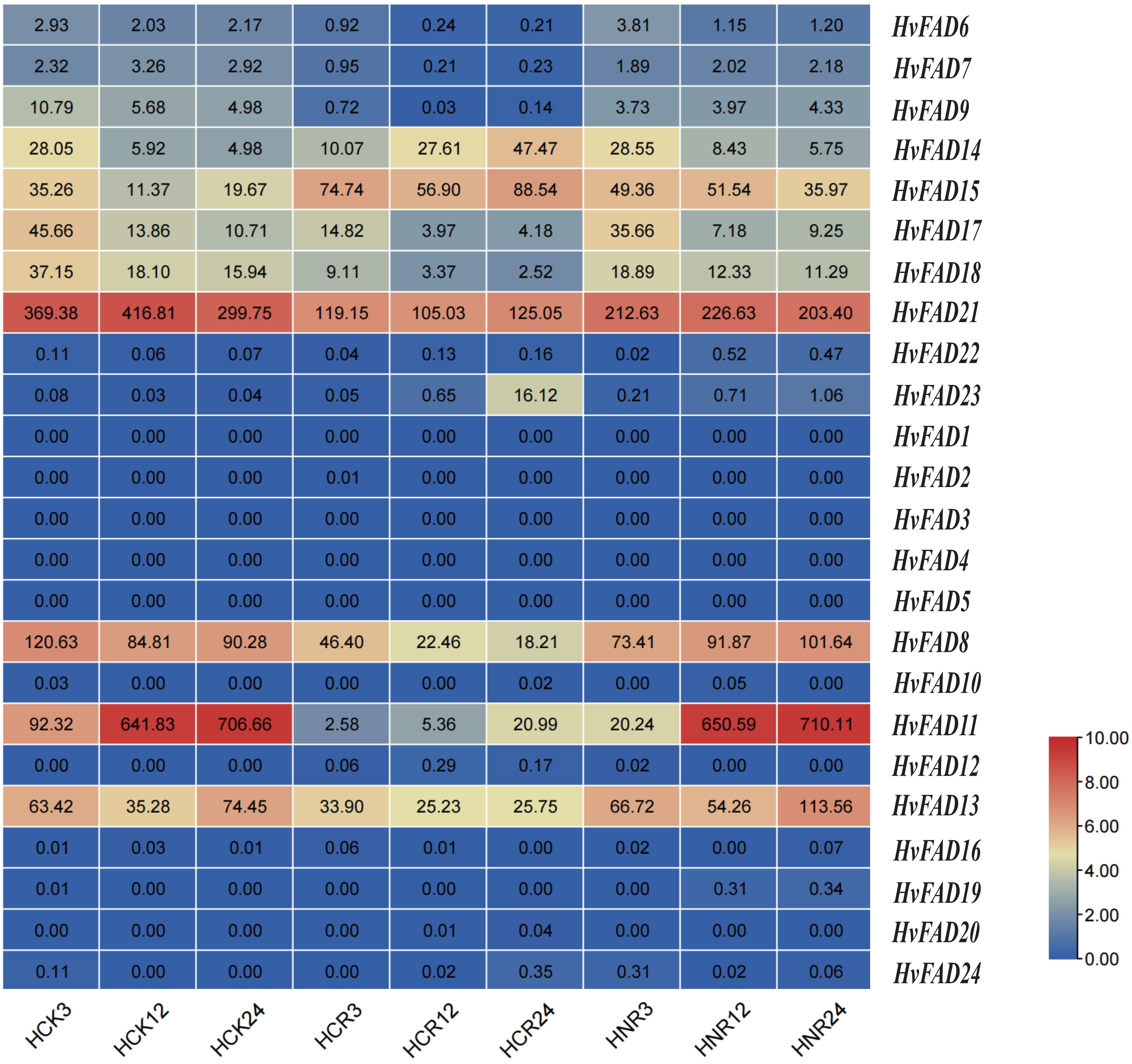
HvFAD6 2.93 2.03 2.17 0.92 0.24 0.21 3.81 1.15 1.20
HvFAD7 2.32 3.26 2.92 0.95 0.21 0.23 1.89 2.02 2.18
HvFAD9 10.79 5.68 4.98 0.72 0.03 0.14 3.73 3.97 4.33
HvFAD14 28.05 5.92 4.98 10.07 27.61 47.47 28.55 8.43 5.75
HvFAD15 35.26 11.37 19.67 74.74 56.90 88.54 49.36 51.54 35.97
HvFAD17 45.66 13.86 10.71 14.82 3.97 4.18 35.66 7.18 9.25
HvFAD18 37.15 18.10 15.94 9.11 3.37 2.52 18.89 12.33 11.29
HvFAD21 369.38 416.81 299.75 119.15 105.03 125.05 212.63 226.63 203.40
HvFAD22 0.11 0.06 0.07 0.04 0.13 0.16 0.02 0.52 0.47
HvFAD23 0.08 0.03 0.04 0.05 0.65 16.12 0.21 0.71 1.06
HvFAD1 0.00 0.00 0.00 0.00 0.00 0.00 0.00 0.00 0.00
HvFAD2 0.00 0.00 0.00 0.01 0.00 0.00 0.00 0.00 0.00
HvFAD3 0.00 0.00 0.00 0.00 0.00 0.00 0.00 0.00 0.00
HvFAD4 0.00 0.00 0.00 0.00 0.00 0.00 0.00 0.00 0.00
HvFAD5 0.00 0.00 0.00 0.00 0.00 0.00 0.00 0.00 0.00
HvFAD8 120.63 84.81 90.28 46.40 22.46 18.21 73.41 91.87 101.64
HvFAD10 0.03 0.00 0.00 0.00 0.00 0.02 0.00 0.05 0.00
HvFAD11 92.32 641.83 706.66 2.58 5.36 20.99 20.24 650.59 710.11
HvFAD12 0.00 0.00 0.00 0.06 0.29 0.17 0.02 0.00 0.00
HvFAD13 63.42 35.28 74.45 33.90 25.23 25.75 66.72 54.26 113.56
HvFAD16 0.01 0.03 0.01 0.06 0.01 0.00 0.02 0.00 0.07
HvFAD19 0.01 0.00 0.00 0.00 0.00 0.00 0.00 0.31 0.34
HvFAD20 0.00 0.00 0.00 0.00 0.01 0.04 0.00 0.00 0.00
HvFAD24 0.11 0.00 0.00 0.00 0.02 0.35 0.31 0.02 0.06
HCK3
HCK12
HCK24
HCR3
HCR12
HCR24
HNR3
HNR12
HNR24
10.00
8.00
6.00
4.00
2.00
0.00
