# Supplementary material for: Genome-wide identification and characterization of FAD family genes in barley
Source: PeerJ. 2024 Feb 29;12:e16812. doi: 10.7717/peerj.16812 (PMC10909363; doi:10.7717/peerj.16812)
Supplement: Supplemental Information 6 [file peerj-12-16812-s006.pdf]

MIQE Checklist

1. Sample collection, processing, and preparation.

1.1 Collection: Barley leaves were treated with NaCI and NaHCO3 for 0, 3, 12, and 24 hours at the two leaf one heart stage. The expression of HvFAD in different tissues was obtained from barley leaves, roots, tillering tissues, and stems at the two leaf one heart stage.

1.2 Treatment: RNA was extracted by freezing fresh tissues with liquid nitrogen and extracting RNA within -80 ℃ for one day.

1.3 Preparation: Take three replicates from each sample, extract RNA, measure RNA concentration, and calculate to ensure that the pre transcriptional RNA content of each sample is 1000ng, thus ensuring sample homogenization.

2. Quality control of nucleic acid.

2.1 RNA extraction: On an unmanned and clean operating test bench, wear laboratory clothes, masks, and gloves. Grind the sample using liquid nitrogen and a frozen rapid ball mill, and then use the RC411 reagent kit from Novozan Biotech Co., Ltd (Beijing, China). The 10 ul, 100 ul, and 1000 ul gun heads from Axigen Company without RANase, and a 1.5ml centrifuge tube without RANase. Perform the entire process on ice and at 4 ℃. After RNA extraction, immediately reverse transcript and use the remaining RNA, and store it at -80 ℃.

2.2 Use Thermo nucleic acid detection spectrophotometer to measure RNA concentration, and the RNA concentration is around 80ng/ul-500ng/ul.

2.3 gDNA removal: When RNA was extracted using the RC411 reagent kit from Novozan Biological Co., Ltd. (Beijing, China), FastPure gDNA Filter Columns III and Buffer RWA were used to remove gDNA twice.

2.4 Quality assessment: In this experiment, RNA quality was evaluated by gel electrophoresis.

3.Reverse transcription.

3.1 Extract RNA using the R333 reagent kit from Novozan Biotechnology Co., Ltd (Beijing, China) Add 4ul 5 × All in one qRT SuperMix, 1ul No RT Control Mix, 1000ngRNA, RNase free ddH2O supplemented to a 20ul system to a 200ul centrifuge tube without RNAase according to the instructions. The PCR reaction was carried out at 50 ℃ for 15 minutes and 85 ℃ for 5 seconds. Using Axigen's 10ul, 100ul, and 1000ul gun heads without RANase and 200ul centrifuge tubes without RANase.

4.-qPCR.

4.1 Gene and primer information.

Gene Number	qRT-PCR	Amplicon Length	Gene_ID
HvFAD8	CAAGGTGCACACGTAGTCCT<br>TGACTACACGGTGCTTGCAT	208bp	HORVU.MOREX.r3.2HG0161410.1
HvFAD13	GGAACTCAGAGAACGTGCCA<br>GGGGCTAGCACCTGTTTCAT	105bp	HORVU.MOREX.r3.3HG0310210.1
HvFAD14	AGCTTCTCGGACAGCTTGAC<br>AGCGTAGCTTCTTGGTGCTT	160bp	HORVU.MOREX.r3.4HG0354970.1
HvFAD15	CCCGTAGTCCCGATCAAGTG<br>TCTGACCTTTGTGATGGGGC	150bp	HORVU.MOREX.r3.4HG0384980.1
HvFAD7	AGTTTGGCGTGTGTCTTTGC<br>CCTGTGCAGCATTCCACTCT	157bp	HORVU.MOREX.r3.2HG0129520.1

4.2 Experimental design: Tublin (F: AGTGTCCTGTCCACCACCTC R: AGCATGAAGTGGATCCCTTGG) was selected as the internal reference primer, and the three repeated cDNA after reverse transcription were mixed and diluted 5-fold. Each sample of each gene was labeled with three replicates on a 96 well plate, with the aim of eliminating sample errors and experimental mechanical errors. Each sample corresponds to both the target gene primer and internal reference primer.

4.3 Reaction system and reaction conditions: The reaction was carried out using the Q712 reagent kit from Novozan Biotechnology Co., Ltd (Beijing, China). The following mixture was added to the qPCR tube:2 × Taq Pro Universal SYBR qPCR Master Mix 10ul,Primer1 (10 μM) 0.4ul,Primer2 (10 μM) 0.4ul,Template cDNA 2ul, ddH2O 7.2 μl.The PCR program was as follows: 94°C for 2 min; 39 cycles of 95°C for 5 s, 60°C for 30 s; 95°C for 5 s, 65°C for 5 s, and 95°C for 5 s. The PCR program was performed as follows.After adding samples to the qPCR special 96 well plate of Axigen company, seal it with the sealing film of Axigen company (extend the edge and press the sealing film, making sure not to touch the film above the hole throughout the entire process).

4.4 Instrument consumables: Axygen's qPCR dedicated 96 well plate and sealing film, bio rad c1000 qPCR instrument.

5.Data analysis.

5.1 Data calculation method and normalization method: Compare the measured

sample with the control sample Δ Substitute the Ct value into formula 2 ^ (- Ct) to calculate the relative content of the target sequence in the sample to be tested.

5.2 software: Bio-Rad CFX Maestro (admin).
